# Rapid PCR-based diagnosis of superficial mycoses with ROC-optimized cutoffs

**DOI:** 10.1128/spectrum.01357-26

**Published:** 2026-08-14

**Authors:** Yuyuan Xue, Mingjing Wei, Yuhang Mei, Xindi Ma, Li Li, Junhao Zhu, Guo Chen, Min Zhu

**Affiliations:** 1 Department of Dermatology, Huashan Hospital, Fudan University 12478, Shanghai, China; Indiana University School of Medicine, Indianapolis, Indiana, USA

**Keywords:** superficial mycoses, multiplex PCR, ROC curve, diagnostic accuracy, combined testing

## Abstract

Fluorescence microscopy and fungal culture are the primary diagnostic methods for superficial fungal infections, but they are limited by low sensitivity and long turnaround time, respectively. We determined the optimal cycle threshold (Ct) cutoff for a novel multiplex PCR assay (iFIND) using receiver operating characteristic analysis and evaluated whether a “microscopy + iFIND” combined strategy improves diagnostic performance. This prospective study enrolled 113 patients with suspected superficial mycoses. Specimens underwent fluorescence microscopy, fungal culture, and automated iFIND. Using a composite reference standard, we calculated positive percent agreement (PPA), negative percent agreement, and overall percent agreement. Cohen’s kappa and McNemar’s test assessed inter-method agreement. To mitigate verification bias, we performed a sensitivity analysis excluding cases diagnosed solely by clinical response. Internal bootstrap validation (1,000 resamples) was used to assess overfitting of the Ct cutoff. The optimal Ct cutoff for any fungal infection was 32.66 (area under the curve [AUC] = 0.746). Bootstrap validation confirmed robustness (optimism-corrected AUC = 0.7431, optimism = 0.00275, bias-corrected and accelerated 95% confidence interval for cutoff: 32.5–33.7). iFIND showed excellent discrimination for *Epidermophyton* (AUC = 0.824) and *Trichophyton* (AUC = 0.834). The combined approach achieved a PPA of 65.6%, superior to microscopy alone (50.0%, *P* < 0.001) and comparable to culture (75.0%, *P* = 0.701), while reducing turnaround time from weeks to <3 h. Sensitivity analysis excluding 15 clinically diagnosed cases yielded consistent results: iFIND alone PPA = 46.9% (23/49), combined strategy PPA = 69.4% (34/49). iFIND detected an additional 17.2% of infections missed by conventional methods. The iFIND assay is a rapid, accurate tool for superficial mycoses. The microscopy + iFIND strategy combines speed and sensitivity, offering culture-comparable utility with substantially faster results. However, positive results should be interpreted in a clinical context to avoid overtreatment.

**IMPORTANCE:**

Fungal skin infections affect one in four people worldwide, but current diagnostic methods are either insensitive (microscopy) or painfully slow (culture, taking 2–4 weeks). This study validates a rapid PCR test that delivers results in under 3 h and, when combined with microscopy, matches the accuracy of culture. By establishing a data-driven cutoff value for test positivity and using rigorous statistical methods to confirm its reliability, we provide a practical framework that clinics can adopt immediately. The combined strategy reduces diagnostic delay, enabling same-day treatment decisions and reducing unnecessary antibiotic use. This is especially critical, given the global spread of antifungal-resistant strains, where rapid genus-level identification can guide appropriate therapy and curb resistance. Our approach offers an actionable, culture-comparable solution for routine clinical practice, directly addressing the urgent need for faster, more accurate fungal diagnostics worldwide.

## INTRODUCTION

Superficial fungal infections are among the most prevalent infectious skin diseases globally, affecting approximately 20%–25% of the population, with higher prevalence in tropical and subtropical regions (1, 2). Although rarely life-threatening, associated symptoms impose substantial psychological and physical burdens. These include pruritus, pain, and cosmetic disfigurement (3). In the United States, ambulatory visits for cutaneous fungal infections totaled 6 million between 2005 and 2016 (0.54% of all outpatient visits), with a significant upward trend (*P* = 0.03) (4), reflecting a growing disease burden (5). While regional variations exist, *Trichophyton rubrum* and *Trichophyton interdigitale* remain the predominant isolates (6–8).

The epidemiological landscape has recently changed with the emergence and global spread of antifungal-resistant strains. Terbinafine-resistant *Trichophyton indotineae*, initially reported in South Asia, has now been identified on six continents, causing recalcitrant tinea corporis and cruris (5). Concurrently, *Trichophyton mentagrophytes* genotype VII (TMVII) has been increasingly reported in France and the United States, often involving oral and perianal regions (5). These developments, coupled with increasing resistance among conventional species, render rapid and accurate diagnosis more urgent than ever (3, 7).

Current clinical diagnosis relies primarily on microscopy and culture. Fluorescence staining (e.g., calcofluor white) has improved sensitivity over conventional KOH mounts and provides results within minutes. However, microscopy remains operator dependent and specimen quality dependent (6, 9, 10) and generally cannot identify pathogens to the genus level (3). Fungal culture, considered a “quasi-gold standard” (1), enables genus-level identification but requires 2–4 weeks of incubation, considerably delaying clinical decisions (3). Culture positivity rates are also suboptimal: a German study reported 23.9% positivity in suspected tinea pedis or onychomycosis (11), and a Singapore study found only 16.7% positivity in skin specimens, with only moderate agreement between microscopy and culture (kappa = 0.487) (6).

Given these limitations, multiplex real-time PCR offers high sensitivity, high specificity, and rapid turnaround time (1, 8). Recent studies have demonstrated that multiplex PCR assays achieve 92.9%–94.6% sensitivity and 96.6% specificity for dermatophyte detection, significantly outperforming culture (3, 8). Importantly, PCR can detect low-burden infections missed by conventional methods (3), offering etiological confirmation for clinically suspected but culture-negative cases.

Despite these advantages, key challenges remain. Most existing studies use qualitative interpretation and lack standardized, clinically validated cycle threshold (Ct) cutoffs derived from empirical data (3). Performance varies across platforms, underscoring the need for platform-specific validation. Furthermore, it remains unclear how best to integrate PCR into clinical workflows, specifically, whether a “microscopy + PCR” combined approach can achieve diagnostic performance comparable to culture while dramatically reducing turnaround time.

This study aims to establish evidence-based Ct cutoff values for a novel multiplex real-time PCR assay (iFIND) using receiver operating characteristic (ROC) curve analysis, validate its diagnostic performance against a composite reference standard, and evaluate whether a combined “microscopy + iFIND” strategy is comparable to fungal culture in diagnostic accuracy while substantially reducing turnaround time. We hypothesize that the iFIND assay, optimized with appropriate Ct cutoffs, will improve diagnostic sensitivity, identify occult infections, and facilitate early and precise management of superficial fungal infections.

## MATERIALS AND METHODS

### Study design and participants

**Fig 1:**
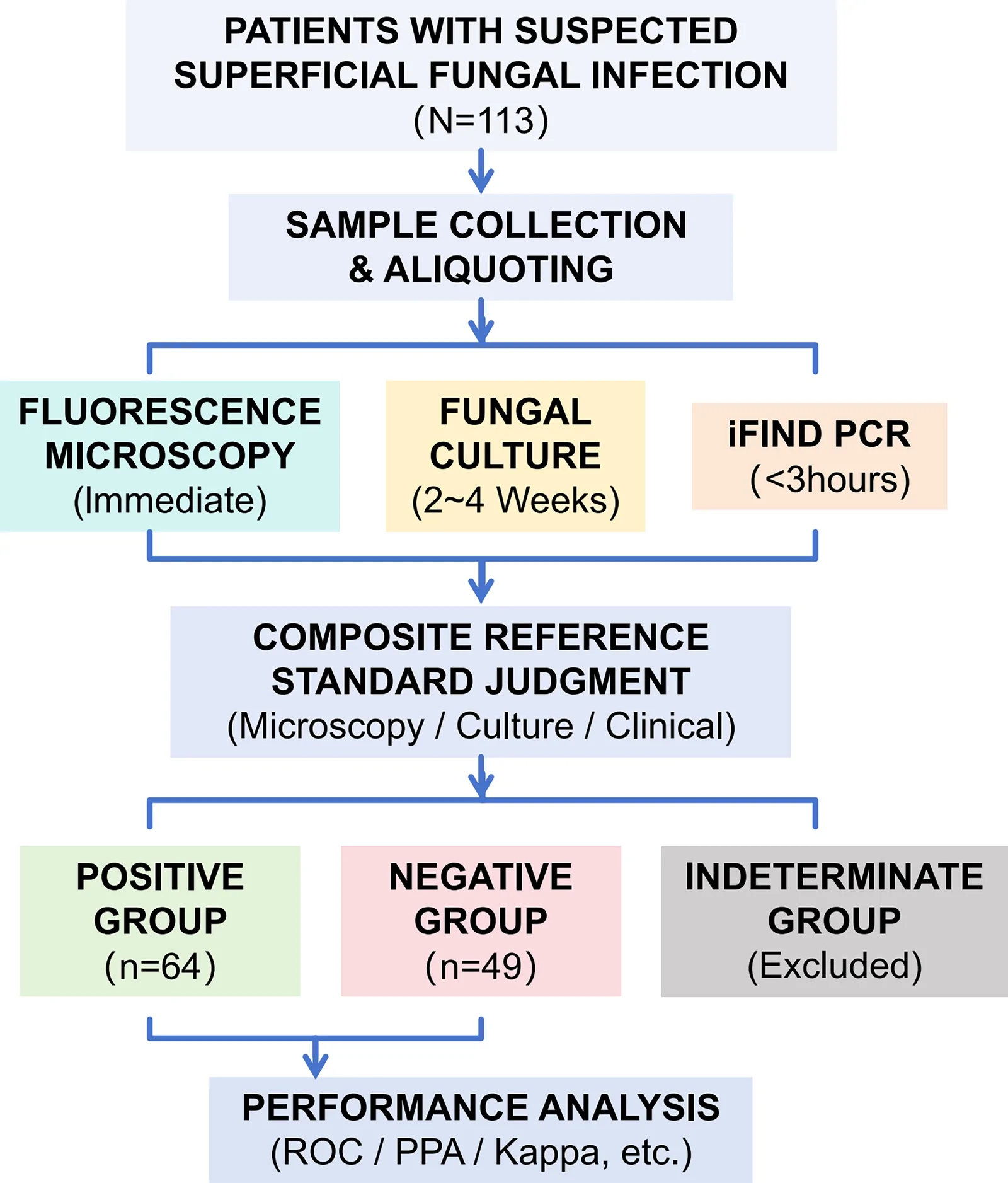
Study flowchart. Flowchart illustrating patient enrollment, specimen collection, parallel testing by fluorescence microscopy, fungal culture, and iFIND PCR assay, composite reference standard determination (including clinical response follow-up), and performance evaluation. The sensitivity analysis excluding clinically diagnosed cases (tier 1, criterion C) is indicated by the dashed box.

This study employed a prospective cohort design, prospectively enrolling patients with clinically suspected superficial fungal infections who presented to the Department of Dermatology, Huashan Hospital, Fudan University, between January 2025 and March 2026. All patients were evaluated by experienced dermatologists, and the decision to perform mycological investigations was based on clinical presentation. Inclusion criteria were patients with typical or atypical skin lesions (e.g., annular erythema, scaling, nail plate thickening, and discoloration) for whom mycological examination was deemed necessary by a dermatologist. Exclusion criteria included use of topical or systemic antifungal agents within the preceding 2 weeks, insufficient specimen quantity to simultaneously perform fluorescence microscopy, fungal culture, and iFIND testing, and refusal to participate in the study. The overall process of the research method is shown in Fig. 1.

### Sample size estimation

The primary endpoint of this study was to verify that iFIND detection in confirmed cases is comparable to fungal culture and to evaluate the incremental clinical value of the combined diagnostic strategy. Sample size calculation was based on international guidelines for diagnostic test studies, using positive percent agreement (PPA) as the core metric.

Based on pilot study data from our center and literature reports, the anticipated PPA of iFIND among culture-positive specimens was assumed to be 85%. With a type I error rate α = 0.05 (two-sided) and an allowable error δ = 0.10, the required sample size for the positive (case) group was calculated using the following formula:


$$N_{pos}=\frac{Z_{1-\alpha /2}^{2}\times PP4\times (1-PP4)}{\delta^{2}}.$$

This yielded an estimated required positive sample size of approximately 49 cases. Based on historical data from our center indicating a fungal culture positivity rate of approximately 42.5% in the pilot phase, the estimated total sample size was calculated as:


$$N_{total}=\frac{49}{0.425}\approx 115\;(\text{cases}).$$

To account for an anticipated 10% exclusion rate due to suboptimal specimen quality, assay failure, or incomplete follow-up data, the target recruitment was set at 130 cases. Ultimately, 113 cases were included in the final analysis, which satisfied the statistical power requirements.

### Detection methods

#### Specimen collection and processing

All specimens were collected by uniformly trained dermatologists or research nurses adhering strictly to standard operating procedures. The collection site was determined by lesion type. For skin infections (tinea corporis, tinea cruris, tinea manuum/pedis), scales were scraped from the active margin of the lesion using a sterile surgical blade. For onychomycosis, subungual debris and discolored or thickened areas of the nail plate were preferentially collected. A sufficient quantity of specimens was obtained from each patient and immediately divided into three equal portions for parallel testing by fluorescence microscopy, fungal culture, and iFIND molecular detection, ensuring that all three methods were performed on homologous specimens.

#### Fluorescence microscopy

One portion of the specimen was placed on a clean glass slide, treated with an appropriate volume of fungal fluorescence staining solution (containing calcofluor white), and covered with a coverslip. After staining at room temperature for 5–10 min, the slide was examined under a fluorescence microscope using excitation light of the appropriate wavelength (340–400 nm). Fungal cell wall chitin and cellulose specifically bind the fluorophore and emit bright blue-green or white fluorescence, whereas background keratin debris appears dark or non-fluorescent. A positive result was defined as the observation of characteristic fungal morphology (hyphae, pseudohyphae, or spores) displaying specific fluorescence. The absence of any fluorescent fungal structures was interpreted as negative. All microscopy results were independently interpreted by two experienced laboratory physicians, with disagreements resolved by consensus. Microscopy operators were blinded to the results of culture and iFIND assays.

#### Fungal culture

One portion of the specimen was inoculated onto Sabouraud dextrose agar slants containing chloramphenicol and cycloheximide and incubated at 25°C–30°C. Cultures were inspected daily starting on day 3 post-inoculation to monitor colony growth, and the time to appearance, morphology, color, and texture were recorded. The incubation period extended to 4 weeks; specimens showing no growth within this period were considered culture negative. For positive cultures, preliminary identification was performed based on macroscopic and microscopic colony morphology (including obverse/reverse pigmentation, texture, and pigment production). For isolates that were difficult to identify morphologically, DNA was extracted, and the internal transcribed spacer (ITS) region was sequenced. Sequences were compared against the GenBank database using BLAST, and isolates with ≥99% homology were identified to the corresponding genus. Culture operators were blinded to the results of microscopy and iFIND assays.

#### iFIND assay

The fully automated nucleic acid detection analyzer (iFIND S2/S4/S8, with throughputs of two/four/eight samples per run, respectively) and the accompanying Superficial Fungal Nucleic Acid Detection Kit (Fluorescence PCR), manufactured by Kunpeng (Xuzhou) Scientific Instrument Co., Ltd., were used. This system enables a fully automated “sample-in, result-out” workflow, integrating ultrasonic lysis, nucleic acid purification, and real-time fluorescence PCR amplification and detection within a single instrument.

##### Specimen pre-processing

Skin scales or nail clippings were placed in specimen processing buffer and vortexed. Two milliliters of the processed sample were loaded into a disposable, fully enclosed cartridge. The cartridge was pre-loaded with ultrasonic buffer, wash buffer, lyophilized beads containing DNA polymerase and primers/probes, and internal control lyophilized beads. The cartridge incorporates multiple contamination-prevention design features, including physical separation of reaction chambers, a unidirectional workflow that prevents cross-contamination between pre- and post-amplification areas, and a sealed cartridge architecture designed to prevent amplicon carryover.

##### Automated run

The cartridge was inserted into the analyzer’s detection unit, and the run was initiated by scanning a QR code. The instrument automatically performed the following steps: (i) ultrasonic lysis (30–60 s for fungal cell disruption); (ii) nucleic acid adsorption and purification via microfiltration membrane; (iii) mixing of purified nucleic acid with lyophilized reagents, with 50 μL of reaction mixture entering the chip-based reaction chamber; (iv) Peltier-based thermal cycling for PCR (ramp rate ≥11.5°C/s, precision ±0.1°C, completed within 30 min); (v) six-channel fluorescence acquisition (internal control: CY55; other dermatophytes: FAM; *Trichophyton* spp.: ROX; *Microsporum* spp.: ATTO425; *Malassezia* spp.: VIC; and *Candida* spp.: CY5); (vi) upon completion, the system output raw Ct values and automated result interpretation.

Of note, dermatophytes encompass *Trichophyton* spp., *Microsporum* spp., and *Epidermophyton* spp. The FAM channel designated as “other dermatophytes” in fact corresponds to *Epidermophyton* spp. Prior to clinical use, the assay was analytically validated at our institution. According to the manufacturer’s technical specifications, the limit of detection was ≤10 CFU/reaction for *Trichophyton rubrum* and *Candida albicans*, with no cross-reactivity observed against common skin commensals (e.g., *Staphylococcus epidermidis* and *Corynebacterium* spp.) or human genomic DNA. These analytical performance metrics support the reliability of the clinical results reported herein. iFIND assay operators were blinded to the results of microscopy and culture.

### Reference standard

A stratified composite reference standard was employed to comprehensively evaluate the diagnostic performance of the iFIND assay and to minimize reference standard bias.

#### Tier 1: reference standard for any fungal infection

This standard was used to evaluate the overall diagnostic performance of iFIND for detecting the presence of “any superficial fungal infection.” A positive reference standard result was defined as a confirmed diagnosis of superficial mycosis, meeting at least one of the following criteria: (i) detection of fungal hyphae or spores morphologically consistent with pathogenic fungi by fluorescence microscopy; (ii) isolation of a fungal colony in culture, confirmed as a pathogenic fungus by ITS sequencing; or (iii) negative results for both microscopy and culture, accompanied by a highly typical clinical presentation. Typical presentations include annular lesions with active, scaling borders for tinea corporis, or nail plate thickening with yellowish-gray discoloration and subungual hyperkeratosis for onychomycosis. Furthermore, the clinical impression must be unexplained by other dermatoses in the absence of antifungal therapy. Finally, definitive and significant improvement must be observed at 4 weeks (±3 days) after a full course of empiric antifungal therapy, as assessed by the same physician at follow-up. A negative reference standard result was defined as the absence of superficial fungal infection, requiring fulfillment of either of the following pathways: Pathway 1 (antifungal therapy given): negative results for both fluorescence microscopy and fungal culture, and no significant improvement after a full course of empiric antifungal therapy (assessed at 4 weeks ± 3 days); or Pathway 2 (alternative diagnosis treated): negative results for both fluorescence microscopy and fungal culture, and significant improvement after a full course of therapy targeting a confirmed alternative non-fungal dermatological diagnosis (e.g., eczema, psoriasis, seborrheic dermatitis, and contact dermatitis), without having received antifungal therapy during the follow-up period (assessed at 4 weeks ± 3 days).

#### Tier 2: genus-specific reference standard

To evaluate the clinical utility of the combined diagnostic strategy, a parallel interpretation rule was prespecified: a positive combined result was defined as a positive result from either test (fluorescence microscopy or iFIND), while a negative combined result was defined as negative results from both tests, and cases where one test was negative and the other result was missing were excluded from the combined analysis. For genus-specific combined analyses, the same “positive if either test positive” rule was applied, with the following specifications: for *Candida* spp. or *Malassezia* spp., microscopy positivity required detection of spores morphologically consistent with the respective genus; for filamentous fungi (*Epidermophyton* spp./*Trichophyton* spp.), microscopy positivity required detection of hyphae (regardless of genus identity).

#### Criteria for the microscopy + iFIND combined diagnostic approach

To evaluate the clinical utility of the combined diagnostic strategy, a parallel interpretation rule was prespecified: a positive combined result was defined as a positive result from either test (fluorescence microscopy or iFIND); a negative combined result was defined as negative results from both tests. Cases where one test was negative and the other result was missing were excluded from the combined analysis. For genus-specific combined analyses, the same positive if either test positive rule was applied, with the following specification: for *Candida* spp. or *Malassezia* spp., microscopy positivity required detection of spores morphologically consistent with the respective genus; for filamentous fungi (*Epidermophyton* spp./*Trichophyton* spp.), microscopy positivity required detection of hyphae (regardless of genus identity).

### Statistical analysis

All data were analyzed using SPSS version 26.0. Categorical data were presented as frequencies and percentages. Continuous data were presented as mean ± standard deviation or median, depending on distribution.

#### Determination of Ct cutoff values

Using the composite reference standard as the state variable and raw Ct values from each iFIND detection channel as the test variable, we constructed ROC curves. The area under the curve (AUC) with its 95% confidence interval (CI) was calculated. The optimal Ct cutoff for each channel was determined by maximizing the Youden index. To avoid bias introduced by arbitrary threshold setting, raw Ct values were treated as continuous variables for ROC analysis without prespecifying any Ct cutoff.

To mitigate the risk of overfitting and to internally validate the stability of the optimal threshold, we performed a bootstrap resampling procedure with 1,000 resamples. Bias-corrected and accelerated (BCa) 95% CIs for the AUC and the optimal Ct cutoff were calculated based on the bootstrap distributions. The optimism of the AUC estimate was also computed to quantify the degree of overfitting.

Furthermore, to address potential verification bias introduced by the composite reference standard (which included clinically diagnosed cases based on treatment response), we conducted a sensitivity analysis by excluding cases that were positive solely by clinical criteria (tier 1, criterion C). The PPA and negative percent agreement (NPA) were recalculated in this restricted cohort to assess the stability of the primary findings.

#### Definition and calculation of diagnostic performance metrics

Given that the composite reference standard employed in this study is not a perfect gold standard, the traditional terms sensitivity and specificity were redefined as PPA and NPA, respectively, to more accurately reflect assay performance. PPA was calculated as *a*/(*a* + *c*), representing the proportion of iFIND-positive results among reference standard-positive samples. NPA was calculated as *d*/(*b* + *d*), representing the proportion of iFIND-negative results among reference standard-negative samples. Overall percent agreement was calculated as (*a* + *d*)/(*a* + *b* + *c* + *d*). These definitions were consistently applied to the comparative analyses of microscopy, culture, and the combined approach against the reference standard.

#### Agreement analysis

Two-by-two contingency tables were constructed comparing each diagnostic method (fluorescence microscopy, fungal culture, iFIND assay, and the microscopy + iFIND combined approach) against the composite reference standard. PPA, NPA, and their 95% confidence intervals were calculated, and Cohen’s kappa coefficient was used to assess agreement between each method and the reference standard, with kappa values interpreted as follows: <0.4 indicated poor to fair agreement; 0.4–0.75 indicated moderate to substantial agreement; and >0.75 indicated excellent agreement. Agreement analyses encompassed two dimensions: first, the diagnosis of any fungal infection, which involved comparison of overall agreement between the four diagnostic approaches and the reference standard; and second, genus-specific diagnosis, which involved assessment of agreement between microscopy, culture, iFIND, and the genus-specific reference standard for the diagnosis of filamentous fungi (*Epidermophyton* spp./*Trichophyton* spp.), *Malassezia* spp., and *Candida* spp.

#### Difference testing

Paired fourfold tables were constructed, and McNemar’s test was used to compare differences in positive detection rates between different diagnostic methods. Comparisons were performed at the following four levels: (i) the combined approach versus microscopy alone for any fungal infection; (ii) the combined approach versus fungal culture for any fungal infection; (iii) the combined approach versus microscopy alone for genus-specific diagnoses (filamentous fungi, *Malassezia* spp., and *Candida* spp.); and (iv) the combined approach versus fungal culture for genus-specific diagnoses. A two-tailed *P* value of <0.05 was considered statistically significant. No adjustment was made for multiple comparisons, given the exploratory nature of genus-specific analyses.

## RESULTS

### Patient baseline characteristics and determination of Ct cutoff values

A total of 113 valid cases were included in the final analysis, including 61 males (54.0%) and 52 females (46.0%), with a mean age of 42.5 years (range, 18–76 years). Based on the composite reference standard, 64 patients were diagnosed with a superficial fungal infection (positive group) and 49 patients were classified as non-infected (negative group). Within the positive group, filamentous fungal infections were identified in 44 cases, which included 40 cases of *Trichophyton* spp., 5 cases of *Epidermophyton* spp., and 1 case of mixed infection (note that individual genus counts sum to more than 44 due to this mixed infection). Additionally, there were 14 cases of *Malassezia* spp. infections and 9 cases of *Candida* spp. infections.

**Fig 2:**
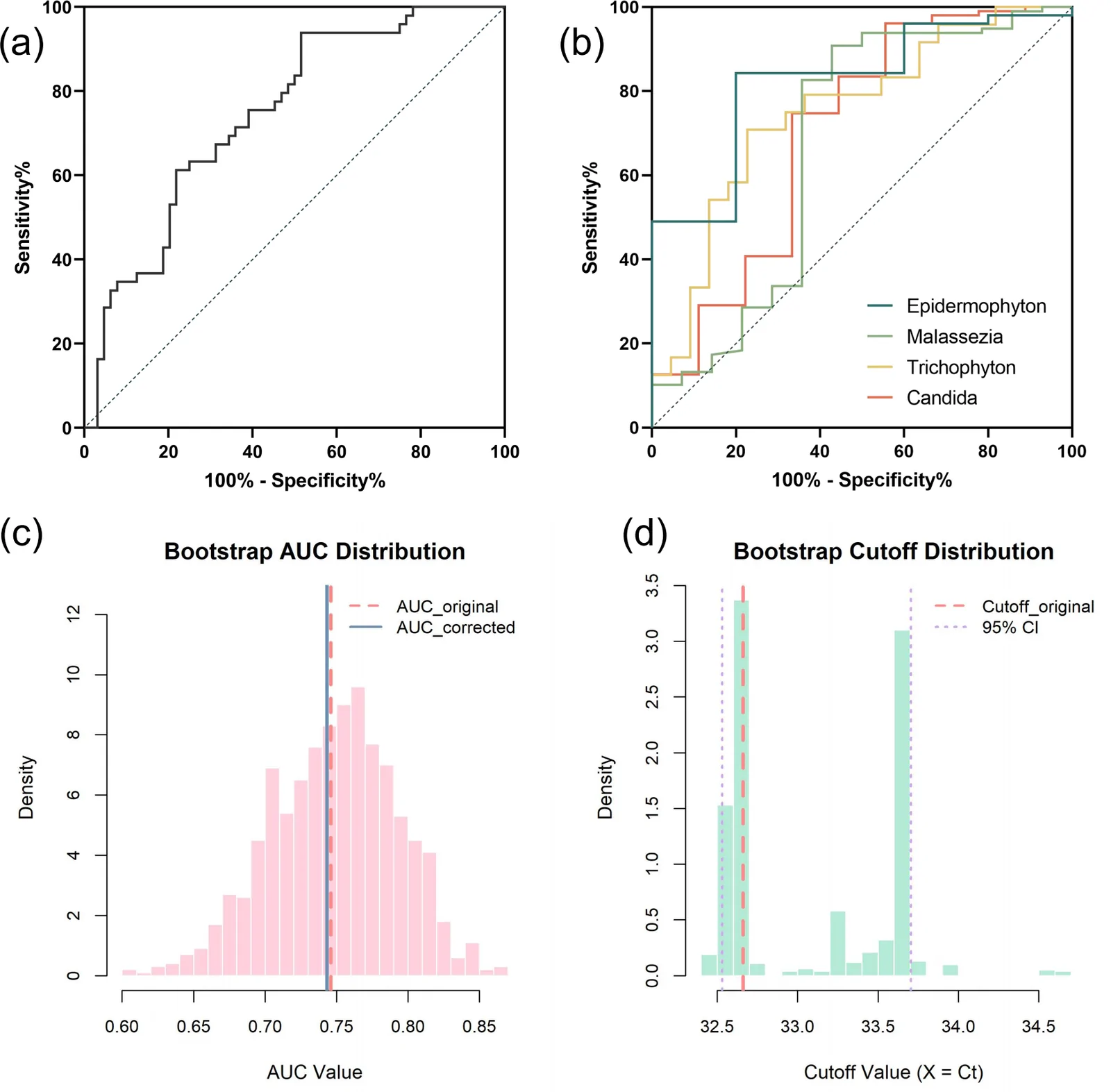
Receiver operating characteristic (ROC) curves and bootstrap validation of iFIND assay. (**a**) ROC curve for any fungal infection (AUC = 0.746, optimal Ct cutoff = 32.66). (**b**) Genus-specific ROC curves for *Epidermophyton* spp. (AUC = 0.824), *Trichophyton* spp. (AUC = 0.834), *Malassezia* spp. (AUC = 0.641), and *Candida* spp. (AUC = 0.704). (**c**) Bootstrap distribution of optimism-corrected AUC (1,000 resamples, optimism = 0.00275). (**d**) Bootstrap BCa 95% CI for the optimal Ct cutoff (32.5-33.7), confirming the robustness of the 32.66 threshold.

ROC curve analysis was performed to determine the optimal Ct cutoff values. Table 1 summarizes the AUCs and cutoffs for each target. The AUC for iFIND in diagnosing any fungal infection (Fig. 2a) was 0.746 (95% CI: 0.656–0.836), and the optimal Ct cutoff value determined by maximizing the Youden index was 32.66. In genus-specific analyses (Fig. 2b), the AUC for *Trichophyton* spp. (*n* = 40) was 0.834 (95% CI: 0.716–0.953), indicating good discriminatory capacity. For pathogens with fewer positive cases, such as *Epidermophyton* spp. (*n* = 5) and *Candida* spp. (*n* = 9), the AUCs were 0.824 (95% CI: 0.653–0.994) and 0.704 (95% CI: 0.491–0.918), respectively; these estimates should be interpreted with caution, given the limited sample sizes. The AUC for *Malassezia* spp. was 0.641 (95% CI: 0.436–0.846). Internal bootstrap validation (1,000 resamples) confirmed the robustness of the derived cutoff. The optimism-corrected AUC was 0.7431 (Fig. 2c), nearly identical to the original AUC of 0.7459 (optimism = 0.00275), and the BCa 95% CI for the optimal Ct cutoff of 32.66 was 32.5–33.7 (Fig. 2d), further supporting its stability.

**TABLE 1:** ROC curve analysis for iFIND in the diagnosis of any fungal infection and genus-specific analyses

Channel	AUC (95% CI)	Optimal Ct cutoff	Youden index	*P* value
Any fungal infection	0.746 (0.656–0.836)	32.66	0.423	<0.001
*Epidermophyton* spp.	0.824 (0.653–0.994)	29.85	0.643	0.018
*Malassezia* spp.	0.641 (0.436–0.846)	31.51	0.470	0.090
*Trichophyton* spp.	0.834 (0.716–0.953)	32.77	0.608	<0.001
*Candida* spp.	0.704 (0.491–0.918)	33.29	0.415	0.043

To further assess the stability of our diagnostic performance estimates, we performed a sensitivity analysis excluding the 15 cases that were diagnosed solely based on clinical response (tier 1, criterion C). In this restricted cohort of 98 patients (49 reference positive and 49 reference negative), the PPA of iFIND alone was 46.9% (23/49), and the PPA of the microscopy + iFIND combined strategy was 69.4% (34/49). These values remained highly consistent with the primary analysis (48.4% and 65.6%, respectively), confirming that our conclusions were not substantially biased by the inclusion of clinically diagnosed cases.

### Diagnostic performance of each method and comparative analysis

Using the composite reference standard as the benchmark, the diagnostic performance metrics for each method are summarized in Table 2. Based on the optimal Ct cutoff of 32.66 determined by ROC analysis, for the diagnosis of any fungal infection, fluorescence microscopy achieved a PPA of 50.0% and an NPA of 100.0%; fungal culture achieved a PPA of 75.0% and an NPA of 100.0%; and the iFIND assay alone achieved a PPA of 48.4% and an NPA of 93.9%. In contrast, the microscopy + iFIND parallel combined strategy achieved a PPA of 65.6%, which was significantly higher than that of microscopy alone (McNemar’s test, *P* < 0.001) and showed no statistically significant difference compared to culture (*P* = 0.701). The combined strategy’s NPA was 93.9%, and the overall percent agreement was 77.9%.

**TABLE 2:** Comparison of diagnostic performance of each method for any fungal infection and genus-specific analyses

Metric	Microscopy	Culture	iFIND	Microscopy + iFIND
Any fungal infection				
PPA (%, 95% CI)	50.0 (37.9–62.1)	75.0 (62.6–84.5)	48.4 (36.6–60.4)	65.6 (52.9–76.5)
NPA (%, 95% CI)	100.0 (92.8–100.0)	100.0 (92.8–100.0)	93.9 (83.5–97.9)	93.9 (83.5–97.9)
Overall agreement (%)	71.7	85.8	68.1	77.9
kappa value	0.464	0.722	0.395	0.569
*Epidermophyton* spp.				
PPA (%, 95% CI)	43.2 (29.7–57.8)^*a*^	100.0 (56.6–100.0)	80.0 (37.6–96.4)	83.3 (68.1–92.1)^*a*^
NPA (%, 95% CI)	100.0 (94.7–100.0)^*a*^	100.0 (96.6–100.0)	84.3 (71.8–92.2)	81.3 (57.0–93.4)^*a*^
Overall agreement (%)	77.9^*a*^	100.0	83.9	82.7^*a*^
kappa value	0.481^*a*^	1.000	0.394	0.614^*a*^
*Trichophyton* spp.				
PPA (%, 95% CI)	43.2 (29.7–57.8)^*a*^	62.5 (47.0–76.0)	73.3 (55.6-85.8)	83.3 (68.1-92.1)^*a*^
NPA (%, 95% CI)	100.0 (94.7–100.0)^*a*^	100.0 (95.0–100.0)	87.5 (64.0–96.5)	81.3 (57.0–93.4)^*a*^
Overall agreement (%)	77.9^*a*^	86.7	78.3	82.7^*a*^
kappa value	0.481^*a*^	0.683	0.559	0.614^*a*^
*Malassezia* spp.				
PPA (%, 95% CI)	64.3 (38.8–83.7)	92.9 (68.5–98.7)	50.0 (26.8–73.2)	64.3 (38.8–83.7)
NPA (%, 95% CI)	100.0 (96.3–100.0)	100.0 (96.3–100.0)	89.8 (82.2–94.6)	89.8 (82.2–94.4)
Overall agreement (%)	95.6	99.1	84.8	86.6
kappa value	0.759	0.958	0.364	0.469
*Candida* spp.				
PPA (%, 95% CI)	44.4 (18.9–73.3)	100.0 (70.1–100.0)	66.7 (35.4–87.9)	66.7 (35.4–87.9)
NPA (%, 95% CI)	100.0 (96.5–100.0)	100.0 (96.5–100.0)	74.8 (65.5–82.4)	74.8 (65.5–82.4)
Overall agreement (%)	95.6	100.0	74.1	74.1
kappa value	0.596	1.000	0.191	0.191

^*a*^ Microscopy cannot reliably distinguish between *Epidermophyton* and*Trichophyton*; therefore, performance metrics are identical for both and are repeated for completeness.

Genus-specific analyses revealed that microscopy had a PPA of 43.2% for filamentous fungi (indicated by hyphae), 64.3% for *Malassezia* spp. spores, and 44.4% for *Candida* spp. spores, with an NPA of 100.0% for all categories. Fungal culture demonstrated excellent performance in genus identification, achieving PPAs of 100.0% for *Epidermophyton* spp. (*n* = 5), 62.5% for *Trichophyton* spp. (*n* = 40), 92.9% for *Malassezia* spp., and 100.0% for *Candida* spp., with an NPA of 100.0% across the board. The iFIND system achieved PPAs of 80.0% for *Epidermophyton* spp., 73.3% for *Trichophyton* spp., 50.0% for *Malassezia* spp., and 66.7% for *Candida* spp., with NPAs ranging from 74.8% to 89.8%. Agreement analyses revealed that microscopy demonstrated moderate to substantial agreement with the reference standard (kappa range: 0.464–0.759); culture demonstrated substantial to excellent agreement (kappa range: 0.683–1.000); and the iFIND assay alone demonstrated fair to moderate agreement (kappa range: 0.191–0.559).

**Fig 3:**
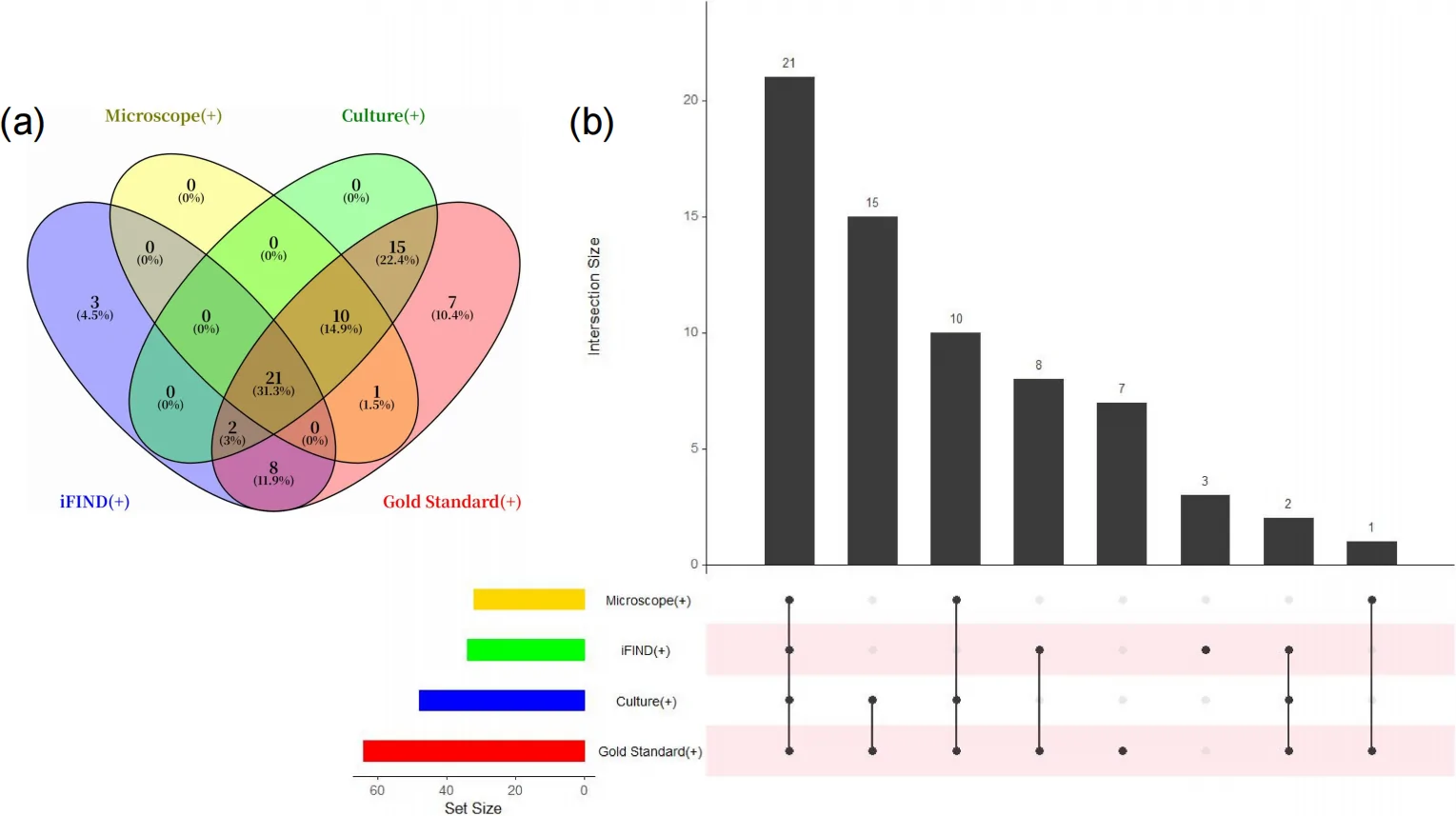
Overlap analysis of positive results among methods. (**a**) Petal Venn diagram showing the overlap of reference standard-positive cases with fluorescence microscopy, fungal culture, and iFIND. Among 64 reference-positive cases, 21 were positive by all three methods; 8 were iFIND-only positive, of which 5 were clinically confirmed (tier 1C) and 3 remained unconfirmed (potential false positives). (**b**) UpSet plot displaying the distribution of positive result combinations. The plot highlights the complementary role of iFIND in detecting cases missed by conventional methods while also illustrating the need for clinical correlation of discordant results.

To further elucidate the overlap among positive results from the three diagnostic methods and the reference standard-positive cases, a three-set Venn diagram (Fig. 3a) and an UpSet plot (Fig. 3b) were constructed. Among the 64 reference standard-positive cases, microscopy detected 32; culture detected 48; and iFIND detected 31 cases that were positive by the reference standard (i.e., true positives). As shown in Fig. 3a, 21 cases (32.8% of reference-standard positives) were positive by all three methods. Ten cases were positive by both microscopy and culture but negative by iFIND; two cases were positive by both culture and iFIND but negative by microscopy; and no cases were positive by both microscopy and iFIND but negative by culture. Fifteen cases were positive by culture only, 8 by iFIND only, and 1 by microscopy only. Of note, 57 of the 64 reference standard-positive cases were detected by at least one method; the remaining 7 cases (10.9%) were negative by all three tests and were diagnosed based on clinical presentation and treatment response (tier 1, criterion C). For comparison, when using fungal culture as the reference (rather than the composite standard), iFIND detected 23 of 48 culture-positive specimens, yielding a PPA of 47.9% (23/48). The UpSet plot (Fig. 3b) further illustrates that iFIND identified eight cases that were negative by both microscopy and culture (iFIND-only positives). Among these, five were true positives according to clinical presentation and treatment response (tier 1, criterion C). The other three cases, however, did not meet any of the composite reference standard criteria. These criteria include microscopy, culture, and clinical response. Therefore, these three cases represent definitive potential false positives. This highlights that although iFIND has superior analytical sensitivity, some positive results may lack clinical or microbiological confirmation, which underscores the need for cautious interpretation and clinical correlation.

### Incremental value analysis of the microscopy + iFIND combined approach versus microscopy alone

Among the 81 specimens that tested negative by microscopy, the microscopy + iFIND combined approach detected an additional 13 positive cases, all of which were confirmed as true positives by the composite reference standard, yielding an additional positivity rate of 16.0% (13/81) among microscopy-negative specimens. This finding suggests that the combined strategy can identify potential occult infections that are often missed by microscopy alone, though all positive results should be interpreted with clinical correlation.

Paired comparisons revealed that the microscopy + iFIND combined strategy significantly increased the number of positive detections compared to microscopy alone across all diagnostic targets, with net gains (i.e., the increase in positive detections) ranging from 10 to 28 cases and zero net losses (Table 3). McNemar’s test indicated statistically significant differences for all comparisons (all *P* < 0.01). Agreement between microscopy and the combined strategy ranged from poor to substantial (kappa range: 0.169–0.748), with the lowest agreement observed for *Candida* diagnosis (kappa = 0.169).

**TABLE 3:** Incremental value of the “microscopy + iFIND” combined approach compared to microscopy alone

Diagnostic target	Net gain (*n*)	Net loss (*n*)	kappa value	McNemar’s test *P* value
Any fungal infection	13	0	0.748	<0.001
Filamentous fungi	14	0	0.498	<0.001
*Malassezia* spp.	10	0	0.599	0.002
*Candida* spp.	28	0	0.169	<0.001

### Comparative analysis of the microscopy + iFIND combined approach versus fungal culture

Using fungal culture as the comparator, the microscopy + iFIND combined approach for diagnosing any fungal infection demonstrated a PPA of 68.8% (33/48), an NPA of 81.5% (53/65), an overall percent agreement of 76.1% (86/113), and a kappa value of 0.507, indicating moderate agreement between the two methods (Table 4). McNemar’s test revealed no significant difference between the two methods (*P* = 0.701). In genus-specific analyses, for filamentous fungi (including *Trichophyton* and *Epidermophyton*), the combined approach achieved a PPA of 88.9% and an NPA of 64.0% against culture; for *Malassezia* spp., the PPA and NPA were 69.2% and 89.0%, respectively. McNemar’s test showed no significant differences for either filamentous fungi or *Malassezia* (*P* > 0.05). For *Candida* spp. (*n* = 9 positive by the composite reference standard), the PPA was 66.7% and the NPA was 74.8%; McNemar’s test indicated a significant difference (*P* < 0.001), suggesting that the combined approach detects *Candida* positivity at a significantly higher rate than culture. However, this finding should be interpreted with caution, given the limited number of *Candida*-positive cases and the possibility that PCR may detect colonization rather than true infection.

**TABLE 4:** Comparative analysis of the “microscopy + iFIND” combined approach versus fungal culture^*a*^

Diagnostic target	PPA (%, *n*/*N*)	NPA (%, *n*/*N*)	Overall agreement (%)	kappa value	McNemar’s test *P* value
Any fungal infection	68.8 (33/48)	81.5 (53/65)	76.1	0.507	0.701
Filamentous fungi	88.9 (24/27)	64.0 (16/25)	76.9	0.534	0.146
*Malassezia* spp.	69.2 (9/13)	89.0 (89/100)	87.5	0.493	0.180
*Candida* spp.	66.7 (6/9)	74.8 (77/103)	74.1	0.191	<0.001

^*a*^ NPA, negative percent agreement using culture as the comparator; PPA, positive percent agreement using culture as the comparator.

## DISCUSSION

Superficial fungal infections represent one of the most common infectious dermatoses worldwide. Rapid and accurate diagnosis is crucial for guiding targeted therapy and mitigating antifungal resistance (1). This study established optimal Ct cutoff values for a novel multiplex iFIND system in diagnosing superficial mycoses using ROC curve analysis and systematically validated its clinical utility. Internal bootstrap validation confirmed the robustness of the derived 32.66 cutoff (optimism-corrected AUC = 0.7431, optimism = 0.00275). The key findings are discussed below in the context of current literature.

Our findings indicate that the iFIND assay alone achieved a PPA of 48.4% and a kappa of 0.395, demonstrating only fair agreement with the reference standard. Superficially, these metrics appear inferior to culture (PPA 75.0%, kappa 0.722) and even microscopy (PPA 50.0%, kappa 0.464). However, this phenomenon is largely attributable to reference standard bias. The composite reference standard, while integrating microscopy, culture, and clinical response, cannot fully circumvent the inherent limitations of conventional methods. Microscopy is operator dependent and cannot detect non-viable fungi (12), whereas culture is constrained by fungal viability and growth rate (3). As a molecular technique, iFIND can detect minute quantities of fungal DNA, even from non-viable organisms, and therefore may yield positive results in conventionally negative specimens. In this study, iFIND detected 11 positive cases among 64 patients with negative microscopy and culture (additional detection rate 17.2%), of which 8 were iFIND-only positives (Fig. 3) and presented as clinically suspicious occult infections. Similarly, Harel et al. reported that most culture-negative but PCR-positive specimens were classified as true positives based on composite clinical criteria (3), and Debuysschere et al. confirmed 28 of 29 such cases by a second PCR method (13). Thus, so-called iFIND “false positives” largely reflect superior analytical sensitivity rather than true misclassifications. However, we must acknowledge a critical distinction: among the eight iFIND-only positive cases (negative by both microscopy and culture), five were classified as true positives based on clinical presentation and treatment response (tier 1, criterion C), but the remaining three cases were negative even by the composite reference standard, meaning they lacked confirmatory evidence from microscopy, culture, and clinical response. These three cases likely represent true false positives, potentially attributable to non-specific amplification, trace contamination, or detection of non-viable fungal DNA without clinical significance. Evaluating molecular diagnostics solely against conventional reference standards may systematically underestimate their true diagnostic value, but conversely, relying solely on molecular results without clinical correlation risks overtreatment.

Our sensitivity analysis, which excluded the 15 cases diagnosed solely by clinical response, yielded nearly identical PPA estimates (iFIND alone: 46.9%, combined strategy: 69.4%), confirming that the inclusion of clinically diagnosed cases did not inflate our performance estimates.

Recognizing that iFIND alone suffers from perceived “underestimation” due to reference standard bias and that microscopy alone has limited sensitivity, we propose a parallel combined microscopy + iFIND strategy. Microscopy provides morphological evidence within minutes (100% specificity), while iFIND delivers sensitive molecular detection and genus-level identification within 3 h, two modalities that complement each other synergistically. Our results robustly support this design. The combined approach achieved a significantly higher PPA (65.6%) than microscopy alone (50.0%; net gain of 13 cases, *P* < 0.001) and showed no statistically significant difference from culture (75.0%, *P* = 0.701). In the sensitivity analysis excluding clinically diagnosed cases, the combined strategy’s PPA was 69.4% (34/49), even closer to the culture's 75.0%, further supporting its clinical utility. Importantly, the combined strategy consistently increased positive detections across all diagnostic targets compared to microscopy alone, with net gains ranging from 10 cases (*Candida*) to 28 cases (any fungal infection) and no net losses (Table 3). This implies that clinicians can obtain culture-comparable diagnostic information on the day of initial consultation, without the 2- to 4-week delay, substantially reducing reliance on empiric therapy. Genus-specific analyses revealed differential performance. For filamentous fungi and *Malassezia*, agreement with culture was satisfactory (McNemar’s *P* > 0.05). However, for *Candida*, the combined approach was significantly more likely to yield a positive result than culture (*P* < 0.001). This discrepancy may reflect that *Candida* can exist as a commensal or colonizer in skin/nail specimens, making it difficult for culture to distinguish colonization from true infection, whereas PCR interpreted in the context of composite clinical criteria may better reflect the actual infectious state (3). Conversely, this also raises the concern that PCR may overcall colonization as infection, potentially leading to overtreatment. Therefore, positive *Candida* results by iFIND should be interpreted with particular caution and correlated with clinical findings, and antifungal therapy should not be initiated solely based on a positive PCR result without supportive clinical or microscopic evidence.

**Fig 4:**
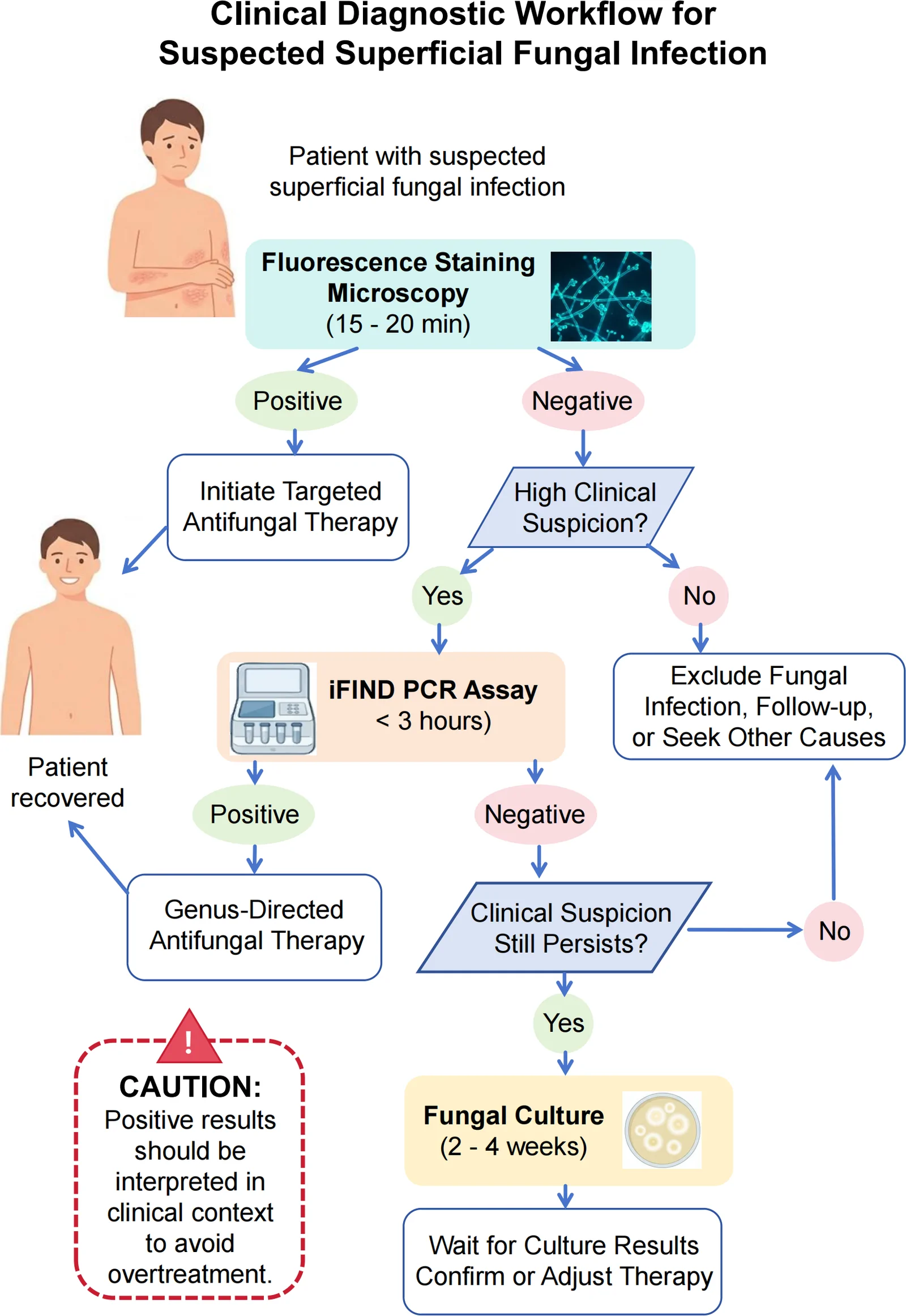
Clinical diagnostic algorithm for superficial fungal infections. For patients with clinically suspected superficial mycoses, fluorescence microscopy (15–20 min) is the first-line screening test. Microscopy-positive patients can initiate targeted antifungal therapy immediately. For microscopy-negative patients, the degree of clinical suspicion guides further steps: iFIND PCR (<3 h) is performed for highly suspicious cases to guide genus-directed therapy. For those with persistent high suspicion despite negative microscopy and iFIND, fungal culture (2–4 weeks) is recommended as a backup confirmatory method. For low-suspicion cases, fungal infection is ruled out. A cautionary note is included: positive iFIND results, particularly for *Candida* spp., should be interpreted in a clinical context to avoid overtreatment.

Turnaround time (TAT) is a critical determinant of clinical decision-making. Conventional culture requires 2–4 weeks, leading to diagnostic delays, reduced adherence, and unnecessary empiric therapy (14). A Finnish study of 14,330 specimens showed that PCR reduced median TAT from 19 days to 16 h (14); Debuysschere et al. reported a median diagnosis time of 15 days for culture versus <7 days for PCR (13). In this study, the fully automated iFIND system required only 3 h from sample loading to result reporting, a >100-fold reduction in TAT compared to culture. Thus, the microscopy + iFIND strategy enables initial screening and confirmatory testing within a single clinic visit, facilitating “same-day diagnosis, same-day treatment.” This is particularly valuable for patients with high clinical suspicion but negative microscopy, obviating follow-up visits and preventing disease progression during the waiting period. From a health economics perspective, although molecular diagnostics may have higher per-test costs, the shortened diagnostic pathway, reduced empiric antifungal use, decreased revisit rates, and improved patient satisfaction may yield overall healthcare savings (1). Tirard-Collet et al. similarly noted that while initial investment in molecular diagnostics is higher, its rapidity and accuracy confer significant advantages in patient management and resource optimization (15). Based on the above findings, we propose a clinical diagnostic decision-making algorithm for superficial fungal infections (Fig. 4) to guide clinical practice. In this algorithm, for patients with negative microscopy and negative iFIND but persistently high clinical suspicion, we recommend concurrent fungal culture as a final backup confirmatory method, given that culture demonstrated the highest PPA (75.0%) in our study, despite its longer turnaround time.

We must emphasize that while the combined strategy enhances sensitivity, it inevitably results in a lower NPA (93.9%) compared to microscopy or culture alone (both 100%). In the era of emerging antifungal resistance, unnecessary antifungal therapy driven by false-positive PCR results must be avoided. Therefore, we recommend that iFIND results, particularly when discordant with microscopy, should always be interpreted in the full clinical context, and treatment decisions should ideally be supported by microscopy positivity or highly typical clinical presentations. The three cases in our study that were positive by iFIND but negative by all reference standard criteria serve as a cautionary reminder that molecular tests, while powerful, are not infallible.

The emergence of antifungal-resistant strains has profoundly shifted the diagnostic paradigm for superficial mycoses. Terbinafine-resistant *Trichophyton indotineae* has now spread across six continents, causing recalcitrant tinea corporis and cruris (5), while TMVII has been increasingly recognized in Europe and North America (5). In this context, a simple binary “yes/no” diagnosis is no longer sufficient; precise genus identification is essential for guiding rational therapy and avoiding empiric treatment failure (16–19). Conventional microscopy cannot reliably identify fungi to the genus level, and culture-based identification is inherently delayed (16, 19). The iFIND system addresses this gap with its six-channel design, enabling simultaneous detection and differentiation of dermatophytes (*Epidermophyton*, *Trichophyton*, and *Microsporum*), *Candida*, and *Malassezia*. Although the current panel does not incorporate resistance gene detection, its rapid genus-level identification provides crucial information for preliminary resistance risk assessment and earlier treatment adjustment (20). The microscopy + iFIND strategy directly meets the contemporary need for rapid, accurate genus-level diagnosis, supporting the precision use of antifungal agents.

This study has several limitations. First, iFIND-positive but culture-negative specimens were not subjected to confirmatory sequencing due to sample volume and budget constraints, which may have led to underestimation of iFIND’s true specificity. Conversely, the inability to sequence-confirm these discrepant results also means we cannot definitively exclude the possibility of false positives, which could lead to overtreatment if results are not interpreted cautiously. Second, this was a single-center study with a modest sample size (113 cases); genus distribution may be region specific, so generalizability warrants caution. Although internal bootstrap validation (optimism = 0.00275) and sensitivity analyses excluding clinically diagnosed cases (which yielded nearly identical PPAs) demonstrated the internal robustness of our findings, external validation in larger, multi-center cohorts is still urgently needed to confirm the transportability of the 32.66 Ct cutoff and the combined diagnostic strategy. Third, some patients may have received prior antifungal therapy, potentially affecting culture positivity rates; however, this bias, if present, actually underscores iFIND’s advantage in detecting low-burden or non-viable fungi. Fourth, the composite reference standard relied on clinical follow-up; cases with incomplete data were excluded, which may have introduced selection bias. Fifth, the *Microsporum* channel (ATTO425) failed to detect any positive signals in this cohort, precluding evaluation for this genus, a technical issue warranting further assay optimization. This limitation does not affect the validity of findings for the other five channels.

### Conclusion

In conclusion, this study established an optimal Ct cutoff of 32.66 for the iFIND assay in diagnosing superficial fungal infections, with excellent discriminatory capacity for *Epidermophyton* spp. and *Trichophyton* spp. (both AUC > 0.82). Bootstrap validation confirmed the robustness of this threshold (optimism = 0.00275). The microscopy + iFIND combined strategy significantly outperformed microscopy alone (PPA 65.6% vs 50.0%, *P* < 0.001; confirmed in sensitivity analysis: 69.4% vs 50.0%) and was comparable to culture (*P* = 0.701) while reducing turnaround time from 2–4 weeks to under 3 h. iFIND also identified an additional 17.2% of infections missed by conventional methods. Based on these findings, we recommend the combined strategy as a first-line approach: microscopy for rapid initial screening, followed by iFIND for microscopy-negative but clinically suspicious cases. However, positive iFIND results should always be interpreted in a clinical context to avoid overtreatment, particularly for *Candida* spp., and fungal culture should be reserved as a backup for cases with persistent high suspicion but negative microscopy and iFIND. Future multicenter studies should incorporate confirmatory sequencing and resistance gene detection to further advance molecular diagnostics.

## Supplementary Material

Reviewer comments

## Data Availability

The raw clinical data, analytical datasets, and ITS sequences of fungal isolates supporting the findings of this study have been deposited in Figshare under DOI 10.6084/m9.figshare.33153176. The ITS sequences (52 sequences in total, accession numbers PZ786076–PZ786127) are also publicly accessible via the NCBI Nucleotide database (https://www.ncbi.nlm.nih.gov/nuccore/). All data are available without restriction.

## SUPPLEMENTAL MATERIAL

OPEN PEER REVIEW reviewer-comments.pdf An accounting of the reviewer comments and feedback.

ASM does not own the copyrights to Supplemental Material that may be linked to, or accessed through, an article. The authors have granted ASM a non-exclusive, world-wide license to publish the Supplemental Material files. Please contact the corresponding author directly for reuse.
