# Supplementary material for: Rapid PCR-based diagnosis of superficial mycoses with ROC-optimized cutoffs
Source: Microbiol Spectr. 2026 Aug 14;14(9):e01357-26. doi: 10.1128/spectrum.01357-26 (PMC13532267; doi:10.1128/spectrum.01357-26)
Supplement: Supplementary file 1 [file reviewer-comments.pdf]

ASM | Microbiology Spectrum

Rapid PCR-Based Diagnosis of Superficial Mycoses with ROC-Optimized Cutoffs

Yuyuan Xue, Mingjing Wei, Yuhang Mei, Xindi Ma, Li Li, Junhao Zhu, Guo Chen, and Min Zhu

Corresponding Author(s): Min Zhu, Fudan University Huashan Hospital

**Review Timeline:**		
	Submission Date:	April 20, 2026
	Editorial Decision:	June 23, 2026
	Revision Received:	July 7, 2026
	Accepted:	July 20, 2026

Editor: Kenneth Gavina

Reviewer(s): The reviewers have opted to remain anonymous.

Transaction Report:

(Note: With the exception of the correction of typographical or spelling errors that could be a source of ambiguity, letters and reports are not edited. The original formatting of letters and referee reports may not be reflected in this compilation.)

DOI: https://doi.org/10.1128/spectrum.01357-26

Re: Spectrum01357-26 (Rapid PCR-Based Diagnosis of Superficial Mycoses with ROC-Optimized Cutoffs)

Dear Dr. Min Zhu:

Thank you for the privilege of reviewing your work. Your manuscript has been selected for resubmission with revisions. Below you will find the instructions from the Spectrum editorial office and the reviewer comments. Please ensure to respond to each of the comments in full.

Please return the manuscript within 60 days; if you cannot complete the modification within this time period, please contact me. If you do not wish to modify the manuscript and prefer to submit it to another journal, notify me immediately so that the manuscript may be formally withdrawn from consideration by Spectrum.

Revision Guidelines
To submit your modified manuscript, log into the submission site at https://spectrum.msubmit.net/cgi-bin/main.plex. Go to Author Tasks and click the appropriate manuscript title to begin. The information you entered when you first submitted the paper will be displayed; update this as necessary. Note the following requirements:

• Upload point-by-point responses to the issues raised by the reviewers in a file named "Response to Reviewers," NOT in your cover letter.
• Upload a compare copy of the manuscript (without figures) as a "Marked-Up Manuscript" file.
• Upload a clean .DOC/.DOCX version of the revised manuscript and remove the previous version.
• Each figure must be uploaded as a separate, editable, high-resolution file (TIFF or EPS preferred), and any multipanel figures must be assembled into one file.
• Any supplemental material intended for posting by ASM should be uploaded with their legends separate from the main manuscript. You can combine all supplemental material into one file (preferred) or split it into a maximum of 10 files with all associated legends included.

For complete guidelines on revision requirements, see our Submission and Review Process webpage. Submission of a paper that does not conform to guidelines may delay acceptance of your manuscript.

Data availability: ASM policy requires that data be available to the public upon online posting of the article, so please verify all links to sequence records, if present, and make sure that each number retrieves the full record of the data. If a new accession number is not linked or a link is broken, provide Spectrum production staff with the correct URL for the record. If the accession numbers for new data are not publicly accessible before the expected online posting of the article, publication may be delayed; please contact production staff (Spectrum@asmusa.org) immediately with the expected release date.

Publication Fees: For information on publication fees and which article types are subject to charges, visit our website. If your manuscript is accepted for publication and any fees apply, you will be contacted separately about payment during the production process; please follow the instructions in that e-mail. Arrangements for payment must be made before your article is published.

ASM Membership: Corresponding authors may join or renew ASM membership to obtain discounts on publication fees. Need to upgrade your membership level? Please contact Customer Service at Service@asmusa.org.

The ASM Journals program strives for constant improvement in our submission and publication process. Please tell us how we can improve your experience by taking this quick Author Survey.

Thank you for submitting your paper to Spectrum.

Sincerely,
Kenneth Gavina
Editor
Microbiology Spectrum

Reviewer #1 (Comments for the Author):

The study "Rapid PCR-Based Diagnosis of Superficial Mycoses with ROC-1 Optimized Cutoffs" by Xue and colleagues outlines the results of a prospective cohort study to assess the clinical performance of a rapid, multiplex PCR platform, iFIND, in combination and independently of conventional diagnostic methods, including fluorescence microscopy in the diagnosis of superficial fungal infection. The authors also describe an ROC analysis of Ct values generated using the iFIND assay. I present the following questions and suggestions for consideration prior to publication:

Minor Comments:

• Throughout the text, the word "species" is used to describe the identifications yielded by the iFIND assay. The data shown, however, reports results to the genus level only. Consider adjusting wording throughout to reflect that genus level data were generated and reported during this study.

Major Comments:

• Was an analytical validation or verification of iFIND performed at your institution to establish limit of detection, analytical specificity, accuracy and precision of each PCR reaction? Alternatively, are these metrics reported by the manufacturer? Consider outlining these metrics to support the data you report on from the clinical study presented here.

• As outlined in lines 372-375 and Fig 3 of the results, 3 specimens were positive by iFIND, and were not positive by the Gold Standard. Does this suggest that the patients did not meet the criteria outlined in lines 205-214? If so, I would recommend discussing the possibility of these identifications representing false positives in your results and discussion? Regarding my comment above, do analytical specificity (internal or manufacturer) data suggest that the assay has a negligible false negative rate and sufficiently low LoD? Consider the possible clinical implications of iFIND false positives in the diagnostic strategy reported in Fig. 4.

• Consider incorporating a culture component in the diagnostic decision-making process outlined in Figure 4. Culture still represented the most accurate/sensitive diagnostic method in your study (Table 2), and could represent a valuable, concurrent and confirmatory method to the diagnostic workflow presented. Especially in cases where microscopy and iFIND yield negative results in a case where clinical presentation is consistent with superficial fungal infection.

• The main narrative presented by this study is the degree of effectiveness of the iFIND demonstrates in combination with additional diagnostic modalities for the identification of superficial fungal infection. I don't think including the ROC analysis adds to the story, nor does it support the overall conclusions presented. Consider, removing this section.

Many thanks for the opportunity to review your work!

Reviewer #2 (Comments for the Author):

Rapid PCR-Based Diagnosis of Superficial Mycoses with ROC-Optimized Cutoffs
Overall assessment:
Well designed prospective diagnostic study addressing an important gap in rapid diagnosis of superficial mycoses. The manuscript is well written and the microscopy and iFIND strategy is clinically relevant. There are some clarification and methodological concerns that would be good to address.
Major comments:
- Reference standard includes microscopy and culture as well as response to antifungal therapy which are comparator tests, which biases performance estimates. I think it would be important to clarify how bias was mitigated and temper conclusions. Sensitivity analyses would be a beneficial addition especially if it excludes clinical response only cases
- There is a risk of overfitting with Ct derived from the same dataset used for performance evaluation. Bootstrapping could help here.
- iFIND alone shows low PPA (48.4%) and only fair agreement ($\kappa = 0.395$) so the statement that PCR outperforms conventional methods is a bit overstated. I think it would be good to include limitations, inhibitions, variability that can all impact sensitivity
- The parallel testing approach of including both culture and iFIND increases PPA but decreases NPA. Clinical implications of lower specificity aren't adequately covered, it may lead to overtreatment (we are already seeing increased resistance), as well as inability to differentiate between colonization and infection for some organisms.
- Small sample size and there are very limited species, so would temper the conclusions. It's really not generalizable.
- Though there were additional detections, not sure all of the additional detections were clinically relevant

Minor comments:
Line 11-14: limited by low sensitivity and long turnaround time reads awkward
Line 19: overall agreement
Line 145-147: seems like a run on sentence
Line 180-181 - specifics on multiple contamination prevention measures would be helpful
Line 253 - 259: Long sentence, hard to follow
Line297-298: run on sentence structure
Line 366-367: clarify sentence

Rapid PCR-Based Diagnosis of Superficial Mycoses with ROC-Optimized Cutoffs

Overall assessment:

Well designed prospective diagnostic study addressing an important gap in rapid diagnosis of superficial mycoses. The manuscript is well written and the microscopy and iFIND strategy is clinically relevant. There are some clarification and methodological concerns that would be good to address.

Major comments:

- Reference standard includes microscopy and culture as well as response to antifungal therapy which are comparator tests, which biases performance estimates. I think it would be important to clarify how bias was mitigated and temper conclusions. Sensitivity analyses would be a beneficial addition especially if it excludes clinical response only cases
- There is a risk of overfitting with Ct derived from the same dataset used for performance evaluation. Bootstrapping could help here.
- iFIND alone shows low PPA (48.4%) and only fair agreement ($\kappa = 0.395$) so the statement that PCR outperforms conventional methods is a bit overstated. I think it would be good to include limitations, inhibitions, variability that can all impact sensitivity
- The parallel testing approach of including both culture and iFIND increases PPA but decreases NPA. Clinical implications of lower specificity aren't adequately covered, it may lead to overtreatment (we are already seeing increased resistance), as well as inability to differentiate between colonization and infection for some organisms.
- Small sample size and there are very limited species, so would temper the conclusions. It's really not generalizable.
- Though there were additional detections, not sure all of the additional detections were clinically relevant

Minor comments:
Line 11-14: limited by low sensitivity and long turnaround time reads awkward
Line 19: overall agreement
Line 145-147: seems like a run on sentence
Line 180-181 – specifics on multiple contamination prevention measures would be helpful
Line 253 – 259: Long sentence, hard to follow
Line297-298: run on sentence structure
Line 366-367: clarify sentence

Response to Reviewers

Manuscript Title: Rapid PCR-Based Diagnosis of Superficial Mycoses with ROC-Optimized Cutoffs

Corresponding Author: Min Zhu

Journal: *Microbiology Spectrum*

Dear Dr. Gavina and Reviewers,

We sincerely thank the editorial office and the expert reviewers for their valuable time, meticulous assessment, and highly constructive feedback on our manuscript. We greatly appreciate the insightful comments, which have significantly helped us improve the scientific rigor, statistical validity, and clinical clarity of our work.

We have carefully addressed each of the concerns raised. Below, we provide a detailed point-by-point response to all reviewer comments. All modifications in the revised manuscript have been highlighted using the **"Track Changes"** function for easy identification. We believe the revised version is substantially strengthened and hope it now meets the standards for publication in *Microbiology Spectrum*.

Reply to Reviewer #1

We are very grateful to Reviewer #1 for the encouraging words and the meticulous review that helped us refine the terminology and clinical framework.

Comment 1 (Minor): *Throughout the text, the word "species" is used to describe the identifications yielded by the iFIND assay. The data shown, however, reports results to the genus level only. Consider adjusting wording throughout to reflect that genus level data were generated and reported during this study.*

Our Response: We fully agree with this crucial distinction. Our assay detects pathogens at the **genus level** (e.g., *Trichophyton* spp., *Candida* spp., *Malassezia* spp.) rather than the species level. We have carefully revised the entire manuscript, replacing all inappropriate uses of "species" with "genus" or "genera" throughout the Abstract, Introduction, Results, Discussion, Keywords, and Table headings. We have also rephrased the relevant conclusions to accurately reflect the taxonomic resolution of the iFIND system.

Comment 2 (Major): *Was an analytical validation or verification of iFIND performed at your institution to establish limit of detection, analytical specificity, accuracy and precision of each PCR reaction? Alternatively, are these metrics reported by the manufacturer? Consider outlining these metrics to support the data you report on from the clinical study presented here.*

Our Response: We appreciate this request for greater analytical transparency. As suggested, we have added a dedicated description summarizing the manufacturer's analytical validation data, which we also verified internally before commencing the clinical study. This addition appears at **Lines 182-185** in the revised manuscript:

"Prior to clinical use, the assay was analytically validated at our institution. According to the manufacturer's technical specifications, the limit of detection (LoD) was ≤10 CFU/reaction for Trichophyton rubrum and Candida albicans, with no cross-reactivity observed against common skin commensals (e.g., Staphylococcus epidermidis, Corynebacterium spp.) or human genomic DNA. These analytical performance metrics support the reliability of the clinical results reported herein."

These data provide a solid analytical foundation that supports the reliability of our clinical performance results.

Comment 3 (Major): *As outlined in lines 372-375 and Fig 3 of the results, 3 specimens were positive by iFIND, and were not positive by the Gold Standard. Does this suggest that the patients did not meet the criteria outlined in lines 205-214? If so, I would recommend discussing the possibility of these identifications representing false positives in your results and discussion? Regarding my comment above, do analytical specificity (internal or manufacturer) data suggest that the assay has a negligible false negative rate and sufficiently low LoD? Consider the possible clinical implications of iFIND false positives in the diagnostic strategy reported in Fig. 4.*

Our Response: This is an excellent and clinically important point. We have carefully re-examined the 8 iFIND-only positive cases (positive by iFIND but negative by both microscopy and culture). Among these, 5 cases were classified as true positives based on clinical presentation and treatment response (Tier 1 Criteria C). However, the remaining 3 cases did not meet any of the composite reference standard criteria, including clinical response, and therefore represent definitive potential false positives.

In the revised manuscript, we have explicitly distinguished these 3 cases in the Results section at **Lines 400-409**:

"The Upset plot (Figure 3b) further illustrates that iFIND identified 8 cases that were negative by both microscopy and culture (iFIND-only positives). Among these, 5 were true positives according to clinical presentation and treatment response (Tier 1 Criteria C). The other 3 cases, however, did not meet any of the composite reference standard criteria. These criteria include microscopy, culture, and clinical response. Therefore, these 3 cases represent definitive potential false positives. This highlights that although iFIND has superior analytical sensitivity, some positive results may lack clinical or microbiological confirmation, which underscores the need for cautious interpretation and clinical correlation."

We have also expanded our Discussion at **Lines 477-488** to address the risk of false positives:

"However, we must acknowledge a critical distinction: among the 8 iFIND-only positive cases (negative by both microscopy and culture), 5 were classified as true positives based on clinical presentation and treatment response (Tier 1 Criteria C), but the remaining 3 cases were negative even by the composite reference standard, meaning they lacked confirmatory evidence from microscopy, culture, and clinical response. These 3 cases likely represent true false positives, potentially attributable to non-specific amplification, trace contamination, or detection of non-viable fungal DNA without clinical significance. Evaluating molecular diagnostics solely against conventional reference standards may systematically underestimate their true diagnostic value, but conversely, relying solely on molecular results without clinical correlation risks overtreatment."

Furthermore, we have revised **Figure 4** (described in response to Comment 4 below) to incorporate a clinical caveat emphasizing that iFIND results should be interpreted in clinical context to avoid overtreatment.

Comment 4 (Major): *Consider incorporating a culture component in the diagnostic decision-making process outlined in Figure 4.*

Our Response: We fully agree with this constructive suggestion. In the revised manuscript, we have added a dedicated branch for patients with negative microscopy, negative iFIND, but persistently high clinical suspicion in the clinical algorithm described at **Lines 538-543**:

"Based on the above findings, we propose a clinical diagnostic decision-making algorithm for superficial fungal infections (Figure 4) to guide clinical practice. In this algorithm, for patients with negative microscopy and negative iFIND but persistently high clinical suspicion, we recommend concurrent fungal culture as a final backup confirmatory method, given that culture demonstrated the highest PPA (75.0%) in our study, despite its longer turnaround time."

The **Figure 4** legend has been updated accordingly to reflect this addition. This ensures that the diagnostic algorithm now fully leverages the complementary strengths of all three modalities (microscopy, iFIND, and culture).

Comment 5 (Major): *I don't think including the ROC analysis adds to the story, nor does it support the overall conclusions presented. Consider, removing this section.*

Our Response: We respectfully acknowledge the reviewer's perspective. While we believe that the ROC analysis provides valuable empirical evidence for establishing an objective *Ct* cut-off (which has been lacking for the iFIND system in the literature), we agree that it should not overshadow the main clinical narrative—the "microscopy + iFIND" combined strategy. To address this concern, we have:

1. Retained the ROC analysis but explicitly stated that the primary clinical utility lies in the combined diagnostic strategy rather than the isolated *Ct* value (see **Lines 454-457** in Discussion: *"This study established optimal Ct cut-off values for a novel multiplex iFIND system in diagnosing superficial mycoses using ROC curve analysis and systematically validated its clinical utility. Internal bootstrap validation confirmed the robustness of the derived 32.66 cut-off..."*).

2. Interpreted the genus-specific AUCs (with limited sample sizes) with appropriate caution in the Results at **Lines 335-339**: *"For pathogens with fewer positive cases, such as Epidermophyton spp. (n=5) and Candida spp. (n=9), the AUCs were 0.824... and 0.704..., respectively; these estimates should be interpreted with caution given the limited sample sizes."*

Reply to Reviewer #2

We are deeply grateful to Reviewer #2 for the highly rigorous methodological critique and the constructive suggestions regarding statistical validation and clinical interpretation. Addressing these concerns has substantially strengthened our manuscript.

Comment 1 (Major): *Reference standard includes microscopy and culture as well as response to antifungal therapy which are comparator tests, which biases performance estimates. I think it would be important to clarify how bias was mitigated and temper conclusions. Sensitivity analyses would be a beneficial addition especially if it excludes clinical response only cases.*

Our Response: We sincerely thank the reviewer for this critical methodological suggestion. To address the potential verification bias introduced by the composite reference standard (particularly the inclusion of clinically diagnosed cases), we performed a comprehensive **sensitivity analysis** by excluding the 15 cases that were positive solely based on clinical response to antifungal therapy (Tier 1 Criteria C).

This analysis was pre-specified in the **Methods** at **Lines 277-282**:

"Furthermore, to address potential verification bias introduced by the composite reference standard (which included clinically diagnosed cases based on treatment response), we conducted a sensitivity analysis by excluding cases that were positive solely by clinical criteria (Tier 1 Criteria C). The Positive Percent Agreement (PPA) and Negative Percent Agreement (NPA) were recalculated in this restricted cohort to assess the stability of the primary findings."

The results are reported in the **Results** at **Lines 345-352**:

"To further assess the stability of our diagnostic performance estimates, we performed a sensitivity analysis excluding the 15 cases that were diagnosed solely based on clinical response (Tier 1 Criteria C). In this restricted cohort of 98 patients (49 reference-positive and 49 reference-negative), the PPA of iFIND alone was 46.9% (23/49), and the PPA of the 'microscopy + iFIND' combined strategy was 69.4% (34/49). These values remained highly

consistent with the primary analysis (48.4% and 65.6%, respectively), confirming that our conclusions were not substantially biased by the inclusion of clinically diagnosed cases."

We have also tempered our conclusions in the **Discussion** at **Lines 489-492**:

"Our sensitivity analysis, which excluded the 15 cases diagnosed solely by clinical response, yielded nearly identical PPA estimates (iFIND alone: 46.9%; combined strategy: 69.4%), confirming that the inclusion of clinically diagnosed cases did not inflate our performance estimates."

Comment 2 (Major): *There is a risk of overfitting with Ct derived from the same dataset used for performance evaluation. Bootstrapping could help here.*

Our Response: We appreciate this highly professional statistical suggestion. Following the reviewer's advice, we performed an **internal bootstrap validation** with 1,000 resamples. This was pre-specified in the **Methods** at **Lines 271-276**:

"To mitigate the risk of overfitting and to internally validate the stability of the optimal threshold, we performed a bootstrap resampling procedure with 1,000 resamples. Bias-corrected and accelerated (BCa) 95% confidence intervals (CIs) for the AUC and the optimal Ct cut-off were calculated based on the bootstrap distributions. The optimism of the AUC estimate was also computed to quantify the degree of overfitting."

The results convincingly demonstrated the robustness of our model and are reported in the **Results** at **Lines 340-344**:

"Internal bootstrap validation (1,000 resamples) confirmed the robustness of the derived cut-off. The optimism-corrected AUC was 0.7431 (Figure 2c), nearly identical to the original AUC of 0.7459 (optimism = 0.00275), and the BCa 95% confidence interval for the optimal Ct cut-off of 32.66 was 32.5–33.7 (Figure 2d), further supporting its stability."

These bootstrap results are also referenced in the **Abstract** at **Lines 26-27** （*"Bootstrap validation confirmed robustness (optimism-corrected AUC = 0.7431; optimism = 0.00275; BCa 95% CI for cut-off: 32.5--33.7)."*）and in the **Discussion** at **Lines 456-458** （ *"Internal bootstrap validation confirmed the robustness of the derived 32.66 cut-off (optimism-corrected AUC = 0.7431; optimism = 0.00275)."*）and **Lines 577-582** （*"Although internal bootstrap validation (optimism = 0.00275) and sensitivity analyses excluding clinically diagnosed cases (which yielded nearly identical PPAs) demonstrated the internal robustness of our findings, external validation in larger, multi-center cohorts is still urgently needed to confirm the transportability of the 32.66 Ct cut-off and the combined diagnostic strategy."*）.

Comment 3 (Major): *iFIND alone shows low PPA (48.4%) and only fair agreement (κ = 0.395) so the statement that PCR outperforms conventional methods is a bit overstated. I think it would be good to include limitations, inhibitions, variability that can all impact sensitivity.*

Our Response: We fully agree with this important clarification. We acknowledge that our original wording was overly assertive. In the revised manuscript, we have **substantially rephrased** the Abstract, Discussion, and Conclusion to reflect that iFIND *alone* demonstrates modest sensitivity, and its true clinical value lies in the **"Microscopy + iFIND" combined parallel strategy**.

Specific revisions include:

- **Abstract (Lines 29-30):** *"The combined approach achieved a PPA of 65.6%, superior to microscopy alone (50.0%, P < 0.001) and comparable to culture (75.0%, P = 0.701)"* — we no longer claim PCR "outperforms" culture.
- **Discussion (Lines 460-463):** *"Our findings indicate that the iFIND assay alone achieved a PPA of 48.4% and a Kappa of 0.395, demonstrating only fair agreement*

with the reference standard. Superficially, these metrics appear inferior to culture (PPA 75.0%, Kappa 0.722) and even microscopy (PPA 50.0%, Kappa 0.464)."

- **Conclusion (Lines 596-598):** *"The 'microscopy + iFIND' combined strategy significantly outperformed microscopy alone (PPA 65.6% vs. 50.0%, P < 0.001; confirmed in sensitivity analysis: 69.4% vs. 50.0%) and was comparable to culture (P = 0.701)"* — we emphasize the **combined strategy** rather than PCR alone.

Comment 4 (Major): *The parallel testing approach of including both culture and iFIND increases PPA but decreases NPA. Clinical implications of lower specificity aren't adequately covered, it may lead to overtreatment (we are already seeing increased resistance), as well as inability to differentiate between colonization and infection for some organisms.*

Our Response: This is a critical clinical safety concern, and we thank the reviewer for raising it. In the revised **Discussion**, we have added a dedicated paragraph at **Lines 544-553** addressing the risk of overtreatment due to false positives:

"We must emphasize that while the combined strategy enhances sensitivity, it inevitably results in a lower NPA (93.9%) compared to microscopy or culture alone (both 100%). In the era of emerging antifungal resistance, unnecessary antifungal therapy driven by false-positive PCR results must be avoided. Therefore, we recommend that iFIND results, particularly when discordant with microscopy, should always be interpreted in the full clinical context, and treatment decisions should ideally be supported by microscopy positivity or highly typical clinical presentations. The 3 cases in our study that were positive by iFIND but negative by all reference standard criteria serve as a cautionary reminder that molecular tests, while powerful, are not infallible."

Additionally, we have specifically addressed the issue of *Candida* colonization versus infection at **Lines 516-520**:

"Conversely, this also raises the concern that PCR may overcall colonization as infection, potentially leading to overtreatment. Therefore, positive Candida results by iFIND should be interpreted with particular caution and correlated with clinical findings, and antifungal therapy should not be initiated solely on the basis of a positive PCR result without supportive clinical or microscopic evidence."

We have also revised our clinical algorithm (**Figure 4**) to emphasize that iFIND results should serve as a guide rather than an absolute indication for therapy.

Comment 5 (Major): *Small sample size and there are very limited species, so would temper the conclusions. It's really not generalizable.*

Our Response: We fully acknowledge this important limitation. We have significantly **strengthened the Limitations paragraph** in the **Discussion at Lines 570-590**, which now reads in full:

"This study has several limitations. First, iFIND-positive but culture-negative specimens were not subjected to confirmatory sequencing due to sample volume and budget constraints, which may have led to underestimation of iFIND's true specificity. Conversely, the inability to sequence-confirm these discrepant results also means we cannot definitively exclude the possibility of false positives, which could lead to overtreatment if results are not interpreted cautiously. Second, this was a single-center study with a modest sample size (113 cases); genus distribution may be region-specific, so generalizability warrants caution. Although internal bootstrap validation (optimism = 0.00275) and sensitivity analyses excluding clinically diagnosed cases (which yielded nearly identical PPAs) demonstrated the internal robustness of our findings, external validation in larger, multi-center cohorts is still urgently needed to confirm the transportability of the 32.66 Ct cut-off and the combined diagnostic strategy. Third, some patients may have received prior antifungal therapy, potentially affecting culture positivity rates; however, this bias, if present, actually underscores iFIND's advantage in detecting low-burden or non-viable fungi. Fourth, the composite reference

standard relied on clinical follow-up; cases with incomplete data were excluded, which may have introduced selection bias. Fifth, the Microsporum channel (ATTO425) failed to detect any positive signals in this cohort, precluding evaluation for this genus, a technical issue warranting further assay optimization. This limitation does not affect the validity of findings for the other five channels."

In addition to expanding the Limitations section, we have also **tempered the interpretation of genus-specific AUC estimates** in the **Results at Lines 335-339**, explicitly stating that estimates for pathogens with fewer positive cases should be interpreted with caution:

"For pathogens with fewer positive cases, such as Epidermophyton spp. (n=5) and Candida spp. (n=9), the AUCs were 0.824 (95% CI: 0.653–0.994) and 0.704 (95% CI: 0.491–0.918), respectively; these estimates should be interpreted with caution given the limited sample sizes."

We have also revised the **Conclusion** to reflect that our findings, while internally robust, require external validation before broad generalization.

Comment 6 (Major): *Though there were additional detections, not sure all of the additional detections were clinically relevant.*

Our Response: We agree with this prudent perspective. We have rephrased the clinical significance of the 17.2% additional detection rate throughout the manuscript:

- **Abstract (Line 33-34):** *"iFIND detected an additional 17.2% of infections missed by conventional methods."* (kept factual, without claiming all were clinically relevant)
- **Results (Lines 416-418):** *"This finding suggests that the combined strategy can identify potential occult infections that are often missed by microscopy alone, though all positive results should be interpreted with clinical correlation."*

- **Discussion (Lines 478-482):** We explicitly distinguished the 5 clinically confirmed iFIND-only positives from the 3 true potential false positives: *" among the 8 iFIND-only positive cases (negative by both microscopy and culture), 5 were classified as true positives based on clinical presentation and treatment response (Tier 1 Criteria C), but the remaining 3 cases were negative even by the composite reference standard, meaning they lacked confirmatory evidence from microscopy, culture, and clinical response."*

This cautious phrasing better reflects the real-world scenario, where molecular positivity must be integrated with clinical judgment to avoid misinterpretation.

Comment 7 (Minor): *Line 11-14: limited by low sensitivity and long turnaround time reads awkward; Line 19: overall agreement; Line 145-147: seems like a run on sentence; Line 180-181 - specifics on multiple contamination prevention measures would be helpful; Line 253 - 259: Long sentence, hard to follow; Line297-298: run on sentence structure; Line 366-367: clarify sentence.*

Our Response: We sincerely apologize for the stylistic and grammatical issues. We have meticulously revised all the sentences and paragraphs identified by the reviewer. Specifically:

- **Line 11-14 (Abstract):** The abstract's opening sentence has been rephrased to *"Fluorescence microscopy and fungal culture are the primary diagnostic methods for superficial fungal infections, but they are limited by low sensitivity and long turnaround time, respectively."* (**Line 12-14**)for improved readability.
- **Line 19 (Abstract):** "agreement" has been clarified as *"overall percent agreement."* (**Line 20-21**)
- **Line 145-147 (Specimen Collection):** The long sentence has been split into three independent sentences for clarity.

- **Line 180-181 (Contamination prevention):** We have added specific details regarding the contamination-prevention features of the iFIND cartridge system, including physical separation of reaction chambers, unidirectional workflow, and sealed cartridge architecture.

- **Line 253-259 (Tier 1 Criteria C):** The nested "with...with...and with..." structure has been split into four independent, clearly stated criteria.

- **Line 297-298 (Difference Testing):** The repetitive structure has been condensed and unified as "(1) X versus Y for Z; (2) X versus Y for Z..." format.

- **Line 366-367 (Upset plot description):** The run-on sentence has been split into two sentences, with added clarification that the 3 non-confirmed iFIND-only positives represent potential false positives.

We have carefully proofread the entire manuscript to ensure clarity and grammatical correctness.

Summary

We believe that the extensive revisions, including the new bootstrap validation, sensitivity analyses, revised clinical algorithm, comprehensive terminology corrections (species → genus), and tempered conclusions, have comprehensively addressed all concerns raised by the reviewers. We are deeply grateful for the opportunity to improve our work and hope that the revised manuscript is now suitable for publication in *Microbiology Spectrum*.

We look forward to your final decision. Please do not hesitate to contact us if further modifications are required.

Sincerely,

Min Zhu, MD, PhD

Department of Dermatology, Huashan Hospital, Fudan University

Email: zhuminhs@fudan.edu.cn

Re: Spectrum01357-26R1 (Rapid PCR-Based Diagnosis of Superficial Mycoses with ROC-Optimized Cutoffs)

Dear Prof. Min Zhu:

I am pleased to share that your manuscript has been accepted, and I am forwarding it to the ASM production staff for publication. Your paper will first be checked to make sure all elements meet the technical requirements. ASM staff will contact you if anything needs to be revised before copyediting and production can begin. Otherwise, you will be notified when your proofs are ready to be viewed.

Data Availability: ASM policy requires that data be available to the public upon online posting of the article, so please verify all links to sequence records, if present, and make sure that each number retrieves the full record of the data. If a new accession number is not linked or a link is broken, provide production staff with the correct URL for the record. If the accession numbers for new data are not publicly accessible before the expected online posting of the article, publication may be delayed; please contact ASM production staff immediately with the expected release date.

Publication Fees: For information on publication fees and which article types have charges, please visit our website. We have partnered with Copyright Clearance Center (CCC) to collect author charges. If fees apply to your paper, you will receive a message from no-reply@copyright.com with further instructions. For questions related to paying charges through RightsLink, please contact CCC at ASM_Support@copyright.com or toll free at +1-877-622-5543. CCC makes every attempt to respond to all emails within 24 hours.

ASM Membership: Corresponding authors may join or renew ASM membership to obtain discounts on publication fees. Need to upgrade your membership level? Please contact Customer Service at Service@asmusa.org.

Embargo Policy: A press release may be issued as soon as the manuscript is posted on the Spectrum Latest Articles webpage. The corresponding author will receive an email with the subject line "ASM Journals Author Services Notification" when the article is available online.

Cover Image Submissions: If you would like to submit a potential Cover Image, please email a file and a short legend to spectrum@asmusa.org. Please note that we can only consider images that (i) the authors created or own and (ii) have not been previously published. By submitting, you agree that the image can be used under the same terms as the published article. Image File requirements: TIF/EPS, 7.5 inches wide by 8.25 inches tall (at least 2,250 pixels wide by 2,475 pixels tall), minimum 300 dpi resolution (600 dpi preferred), RGB, and no figure elements, e.g., arrows or panel labels. The legend should be a short description of the image, 1-2 sentences recommended. Please download and use this interactive template in Adobe the https://journals.asm.org/pb-assets/html/asm-cover-sizing-tool.html to ensure that your proposed cover image meets our size requirements (https://journals.asm.org/pb-assets/pdf-text-excel-files/ASM-Interactive-Sizing-Cover-Template-1715689791.pdf).

The ASM Journals program strives for constant improvement in our submission and publication process. Please tell us how we can improve your experience by taking this quick Author Survey.

Thank you for submitting your paper to Spectrum.

Sincerely,
Kenneth Gavina
Editor
Microbiology Spectrum
